# Data on optimization of production parameters on *Persea Americana* (Avocado) plant oil biodiesel yield and quality

**DOI:** 10.1016/j.dib.2018.08.064

**Published:** 2018-08-28

**Authors:** Anawe A.L. Paul, Folayan J. Adewale

**Affiliations:** a Department of Petroleum Engineering, College of Engineering, Covenant University, Ota, Nigeria; b Department of Petroleum Engineering, University of Ibadan, Nigeria

**Keywords:** ASTM, Avocado Oil, Biodiesel quality, Biodiesel yield, Transesterification reaction

## Abstract

Biodiesel has continued to receive enormous patronage from world energy demand as a result of its renewable nature, low toxicity, rapid degradation, robust fuel performance and low emission characteristics and its overall environmental friendliness.

Hence, these data showed the optimization of temperature, catalyst concentration and type of catalyst, alcohol type and alcohol to oil molar ratio and reaction time on Avocado plant oil biodiesel yield and quality produced via alkali-catalyzed transesterification reaction. Data on the quality of the biodiesel produced by using the American Standard for Testing Materials (ASTM) procedures for biodiesel characterization with different concentrations of alcohol and catalyst under varying temperatures and reaction durations are also provided. The tested biodiesel properties are the cold flow properties (pour point and cloud point) and the critical parameters such as kinematic viscosity at 40 °C, specific gravity at 15 °C, flash point, cetane number, calorific value, iodine value, acid number and sulphated ash percentage.

**Specifications Table**

**Table:** 

Subject area	Chemical Engineering
More specific subject area	Bio engineering
Type of data	Tables, figures
How data was acquired	Experimental. Avocado biodiesel was synthesized in the laboratory by using the following apparatus: measuring cylinder, Erlenmeyer flask, digital weighing balance, magnetic stirrer, electric oven and separating funnel. The biodiesel qualities were evaluated by using the American Standard for Testing Materials (ASTM) procedures.
Data format	Raw, Analyzed
Experimental factors	Biodiesel yield and quality from transesterification reaction is dependent on a lot of factors. These are reaction time, type of alcohol and alcohol to oil molar ratio, reaction temperature and pressure, concentration and type of catalyst, water content and free fatty acid level in fats and oils.
Experimental features	The Avocado biodiesel was produced in the laboratory by the reaction of a known quantity (1000 ml and 600 ml) of Avocado Oil with selected alcohols (Methanol and Ethanol) at various Alcohol to Oil molar ratios under different operating conditions of temperature and catalyst concentration (NaOH and KOH catalyst) in a process known as trans-esterification reaction.
Data source location	Research Laboratory, PTI, Nigeria.
Data accessibility	Data are available within this article
Related Research Article	None

**Value of the data**

• The data showed the technical viability and environmental friendliness potential of Avocado Oil as new discovery and promising candidate in the endless search for biodiesel fuel.
• The data will provide useful information to the scientific community on the effect of various production parameters on Avocado (*Persea Americana)* plant oil in biodiesel yield and quality produced via alkali catalyzed transesterification reaction.
• The data describes the optimum conditions under which biodiesel production from Avocado Oil can be termed efficient and thereby reducing cost of production and waste.
• The data analyzed and compared the quality of biodiesel obtained by varying degree of alcohol and type of alcohol as well as catalyst concentration and type of catalyst by using American Society for Testing Materials (ASTM) standard procedures and techniques.
• The data will serve as guide to various bio-energy operators on the consequence of increasing and reducing one parameter or the other on biodiesel cold flow properties and critical parameters.

## 1 Data

The data obtained from this research work come from the experimental investigation of the effect of temperature, catalyst concentration and type of catalyst, alcohol type and alcohol to oil molar ratio and reaction time on Avocado plant oil biodiesel yield and quality produced via alkali catalyzed transesterification reaction. These parameters were varied at reasonable ratios and concentrations and the emerging result on biodiesel yield and quality were measured. Table 1and Fig. 2 showed the effect of temperature on Avocado plant oil biodiesel yield production by using Avocado Oil volume of 1000 ml, Methanol to Oil molar ratio of 6:1 and NaOH catalyst concentration of 1%w/w and reaction time of two hours (2 h). Table 2 and Fig. 3 described the effect of type of alcohol and alcohol to oil molar ratio on biodiesel yield produced by reacting 600 ml of Avocado Oil with different concentrations of methanol and ethanol in the presence of 1%w/w concentration of sodium hydroxide catalyst and reaction temperature of 65 °C for two hours. The data on the effect of catalysts type and concentration are presented in Table 3 and Fig. 4. The analyzed biodiesel was produced by reacting 1000 ml of Avocado Oil with NaOH and KOH catalysts at different concentrations. The catalysts were firstly dissolved in methanol to form Alkoxides and methanol to oil molar ratio of 6:1 was used while the transesterification reaction was carried out at temperature of 65 °C and reaction time of two hours. Similarly, Table 4 and Fig. 5 represent the effect of different reaction durations on biodiesel yield by the transesterification of 600 ml of Avocado Oil in the presence of sodium hydroxide catalyst (1%w/w NaOH). The reaction temperature was 65 °C and alcohol to oil molar ratio of 6:1 and 9:1 was used for methanol and ethanol respectively.

**Table 1:** Effect of temperature variation on Avocado biodiesel yield.

**Experiment run**	**Temperature (°C)**	**Biodiesel produced (ml)**	**Biodiesel yield (%)**
1	25	482	48.20
2	30	516	51.60
3	35	550	55.50
4	40	621	62.10
5	45	718	71.80
6	50	792	79.20
7	55	886	88.60
8	60	945	94.50
9	65	962	96.20
10	70	720	72.00

**Fig. 1:**
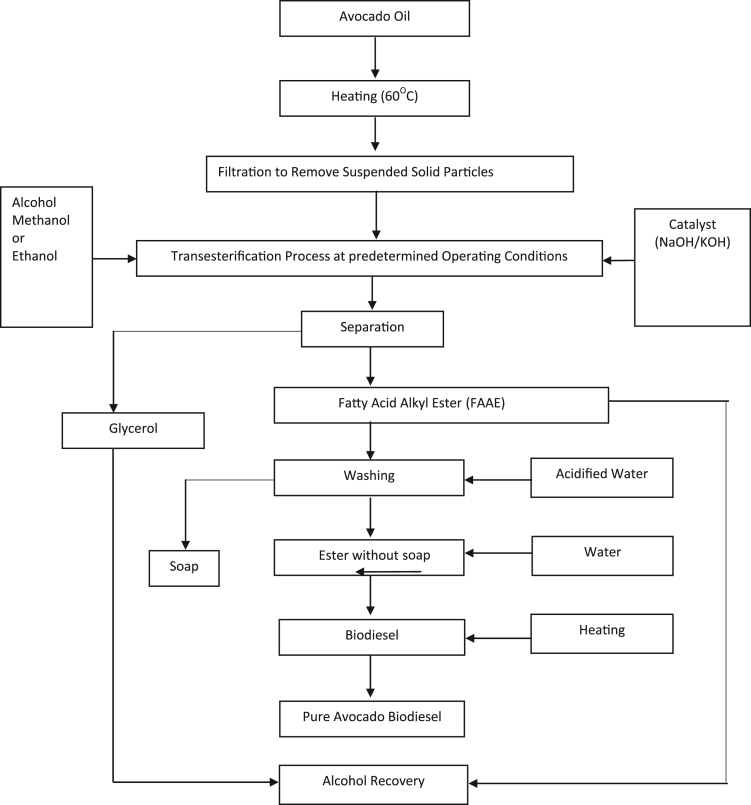
Flow chart for biodiesel production from Avocado plant Oil with < 2.50% Free Fatty Acid.

**Fig. 2:**
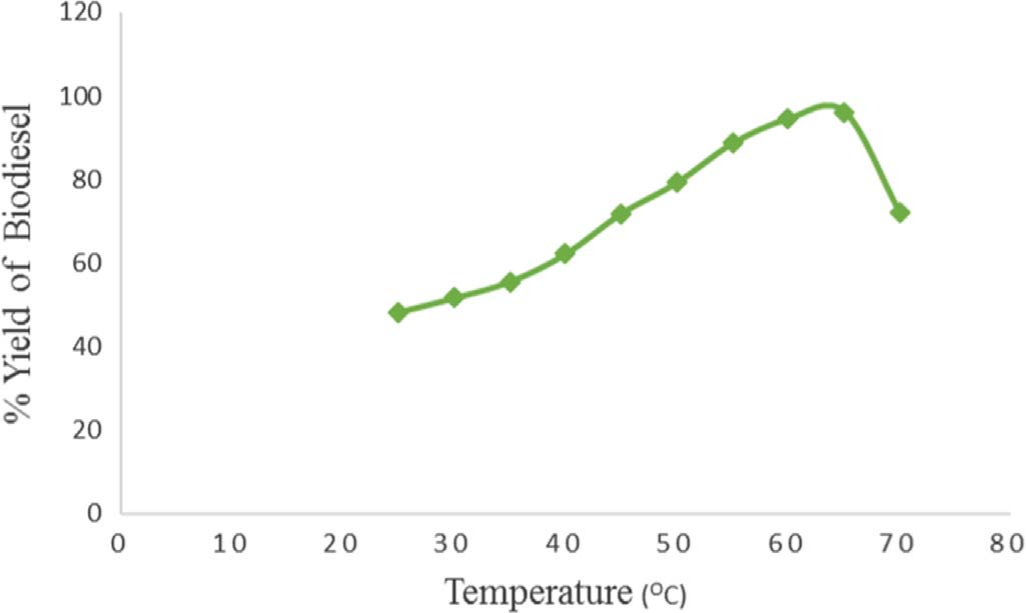
Effect of temperature variation on Avocado biodiesel yield.

**Table 2:** Effect of type of alcohol and alcohol to oil molar ratio variation on Avocado biodiesel yield.

**Experimental run**	**Alcohol:oil**	**Methanol biodiesel produced(ml)**	**Ethanol biodiesel produced(ml)**	**Methanol biodiesel yield(%)**	**Ethanol biodiesel yield(%)**
1	1:1	219	190	36.50	31.67
2	2:1	271	225	45.15	37.50
3	3:1	350	314	58.33	52.33
4	4:1	404	357	67.33	59.50
5	5:1	513	385	85.50	64.17
6	6:1	579	428	96.50	71.33
7	7:1	519	493	86.50	82.17
8	8:1	488	532	81.33	88.67
9	9:1	447	557	74.50	92.83
10	10:1	410	550	68.33	91.67
11	11:1	369	507	61.50	84.50
12	12:1	315	460	52.50	76.67

**Fig. 3:**
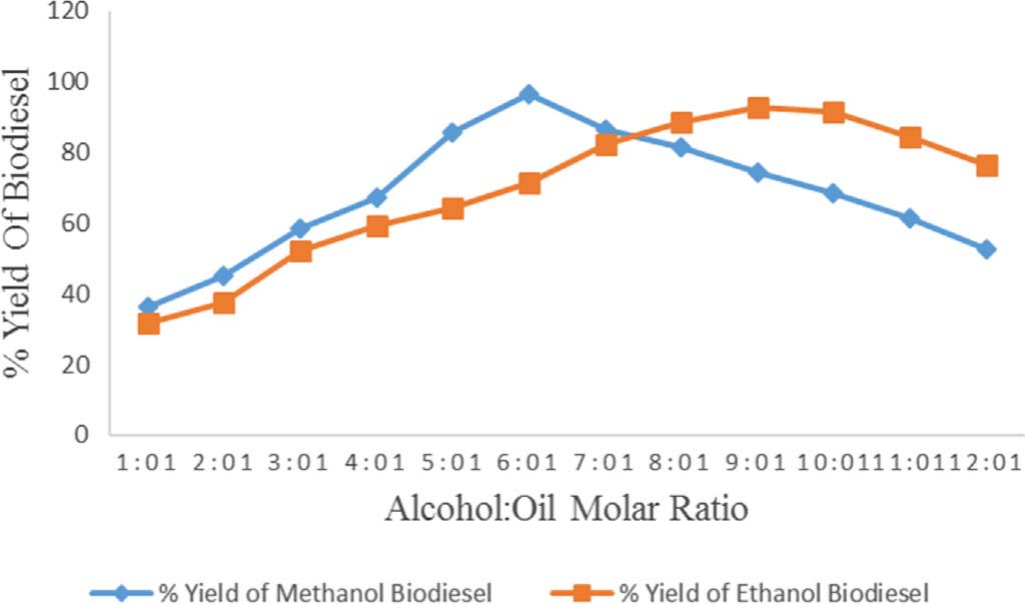
Effect of type of alcohol and alcohol to oil molar ratio on Avocado biodiesel yield.

**Table 3:** Effect of catalyst concentration and type of catalyst on Avocado biodiesel yield.

**Experimental run**	**Catalyst concentration (%w/w)**	**Biodiesel produced(ml) (NaOH catalyst)**	**Biodiesel produced(ml) (KOH catalyst)**	**Biodiesel yield (%) (NaOH catalyst)**	**Biodiesel yield (%) (KOH catalyst)**
1	0.25	341	286	34.10	28.60
2	0.50	565	484	56.50	48.40
3	0.75	865	691	86.50	61.30
4	1.00	941	766	94.10	76.60
5	1.25	845	883	84.50	88.30
6	1.50	755	954	75.50	95.40
7	1.75	630	911	63	91.10
8	2.00	516	788	51.60	78.80

**Fig. 4:**
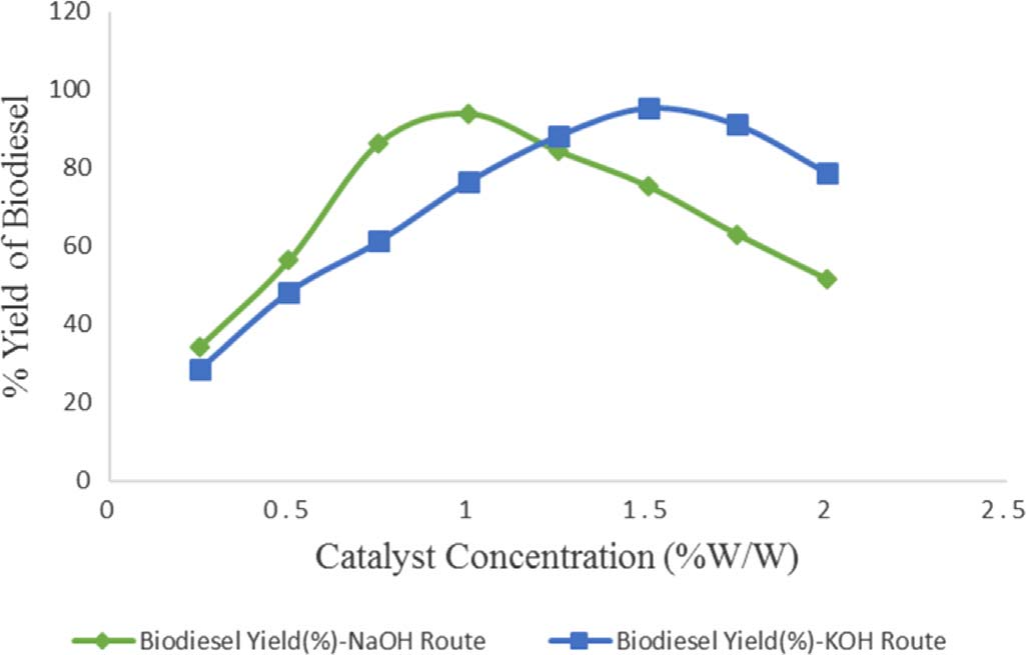
Variation of catalyst concentration and type of catalyst on Avocado biodiesel yield.

**Table 4:** Effect of reaction time variation on Avocado biodiesel yield.

**Experimental run**	**Reaction time (min)**	**Methanol biodiesel produced(ml)**	**Ethanol biodiesel produced(ml)**	**Methanol biodiesel yield (%)**	**Ethanol biodiesel yield(%)**
1	15	231	214	38.50	35.67
2	30	338	306	56.33	51
3	45	411	379	68.50	63.17
4	60	526	471	87.67	78.50
5	75	553	518	92.17	86.33
6	90	567	549	94.50	91.50
7	105	576	554	96	92.33
8	120	580	562	96.67	93.67

**Fig. 5:**
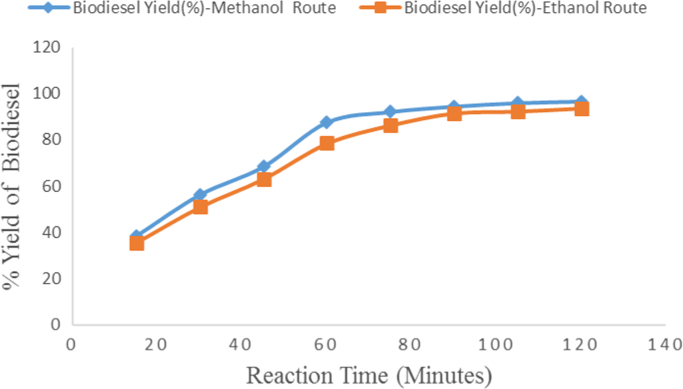
Effect of reaction time variation on Avocado biodiesel yield.

Finally, the effects of various parameters and reagents on Avocado biodiesel qualities are presented in Table 5, Table 6, Table 7, Table 8. The importance of repeatability in data accuracy and precision was considered during the course of the experiment.

**Table 5:** Avocado biodiesel quality characteristics at 1% w/w NaOH, 65 °C reaction temperature, 2 h reaction time and Methanol to Oil ratio of 6:1.

**Test property**	**Test method**	**Test result**	**ASTM standard**
Kinematic viscosity @ 40 °C(mm^2^/s)	ASTM D445	3.75	1.9–6.0
Specific Gravity @ 15 °C	ASTM D1298	0.875	0.86–0.90
Flash Point (°C)	ASTM D93	148	130 min.
Pour Point (°C)	ASTM D5853	− 6	− 10 max.
Cloud Point (°C)	ASTM D2500	4	Report
Cetane Number	ASTM D613	55.10	47 min.
Calorific Value kj/kg	ASTM D240	40,106	Sufficiently close to diesel
Iodine Value $\frac{{\mathrm{gI}}_{2}}{100g\;\mathrm{of}\;\mathrm{biodiese}}$	ASTM D445	78	Report
Acid Number $(\frac{\mathrm{mgKOH}}{g})$	ASTM D664	0.35	0.8max
Sulphated Ash(%)	ASTM D 874	0.016	0.02 max.

**Table 6:** Avocado biodiesel quality characteristics at 1% w/w NaOH, 65 °C temperature, 2 hrs reaction time and Ethanol to Oil ratio of 9:1.

**Test property**	**Test method**	**Test result**	**ASTM standard**
Kinematic viscosity @ 40°C(mm^2^/s)	ASTM D445	4.60	1.9–6.0
Specific Gravity @ 15 °C	ASTM D1298	0.894	0.86–0.90
Flash Point (°C)	ASTM D93	162	130 min.
Pour Point (°C)	ASTM D5853	− 12	− 10 max.
Cloud Point (°C)	ASTM D2500	− 2	Report
Cetane Number	ASTM D613	56.80	47 min.
Calorific Value kj/kg	ASTM D240	40,365	Sufficiently close to diesel
Iodine Value $\frac{{\mathrm{gI}}_{2}}{100g\;\mathrm{of}\;\mathrm{biodiese}}$	ASTM D445	74	Report
Acid Number $(\frac{\mathrm{mgKOH}}{g})$	ASTM D664	0.30	0.80max
Sulphated Ash(%)	ASTM D 874	0.018	max.

**Table 7:** Avocado biodiesel quality characteristics at different NaOH catalyst concentrations using Ethanol to oil ratio of 9:1, temperature of 65 °C and 2 h reaction time.

**Test property**	**0.25% NaOH**	**0.50% NaOH**	**0.75% NaOH**	**1.00% NaOH**	**1.25% NaOH**	**1.50% NaOH**
Kinematic viscosity@ (mm^2^/s)	9.80	8.36	6.94	4.60	4.78	5.86
Specific Gravity @ 15 °C	0.908	0.898	0.891	0.894	0.896	0.905
Flash Point (°C)	186	174	166	162	146	131
Pour Point (°C)	3	− 1	− 6	− 12	2	4
Cloud Point (°C)	9	5	1	− 2	3	6
Cetane Number	48.60	51.50	54.40	56.80	53.60	49.50
Calorific Value kj/kg	39,864	40,110	40,218	40,365	40,146	40,020
Iodine Value $\frac{{\mathrm{gI}}_{2}}{100g\;\mathrm{of}\;\mathrm{biodiese}}$	88	85	81	74	76	83
Acid Number $(\frac{\mathrm{mgKOH}}{g})$	0.84	0.73	0.55	0.30	0.34	0.30
Sulphated Ash(%)	0.048	0.031	0.024	0.018	0.021	0.021

**Table 8:** Avocado biodiesel quality characteristics at different Methanol to Oil ratios using 1%w/w NaOH catalyst, reaction temperature of 65 °C and 2 h reaction duration.

**Test property**	**2:1**	**4:1**	**6:1**	**8:1**	**10:1**	**12:1**
Kinematic viscosity@ (mm^2^/s)	10.65	8.95	3.75	5.88	6.75	8.44
Specific Gravity @ 15 °C	0.912	0.896	0.875	0.886	0.891	0.900
Flash Point (°C)	181	165	148	156	171	186
Pour Point (°C)	8	1	− 6	2	3.5	4
Cloud Point (°C)	12	7	4	5.5	6	10
Cetane Number	47.40	52.60	55.10	53.60	51.80	48.20
Calorific Value kj/kg	39,718	40,036	40,106	40,071	40,110	39,664
Iodine Value $\frac{{\mathrm{gI}}_{2}}{100g\;\mathrm{of}\;\mathrm{biodiese}}$	91	80	78	81	86	89
Acid Number $(\frac{\mathrm{mgKOH}}{g})$	0.95	0.48	0.35	0.28	0.21	0.16
Sulphated Ash(%)	0.061	0.053	0.016	0.044	0.038	0.030

## 2 Experimental design, materials and methods

The Avocado biodiesel was produced in the laboratory by a process known as transesterification [1], [2], [3], [4], [5], [6], [7] and the reaction is a six staged process of oil heating, formation of alkoxide, transesterification reaction, products separation, crude biodiesel purification and methanol recovery. The process flow chart is shown in Fig. 1. The process involves the reaction of a known quantity (1000 ml and 600 ml) of Avocado Oil with selected alcohols (Methanol and Ethanol) at various Alcohol to Oil molar ratios under different operating conditions of temperature and catalyst concentrations (NaOH and KOH catalyst). The reaction mechanism has three main steps as shown in Eqs. (1), (2), (3). The first step involves an attack by the alkoxyde ion, to form a tetrahedrical intermediate. The second step involves the generation of alkoxyde by the reaction between the intermediate produced in the first step with an alcohol molecule. While in the last step, ester and diglycerides are produced with the liberation of three ester molecules(biodiesel) and glycerol (propane 1,2,3 triol).

Reaction mechanism

(1) $$\mathrm{Step}1\;\mathrm{Triglycerides}+{\mathrm{CH}}_{3}\mathrm{OH}\frac{\mathrm{catalyst}}{\mathrm{Temperature}}\to \mathrm{Di}-\mathrm{glycerides}+\mathrm{Methyl ester}$$

(2) $$\mathrm{Step}2\;Di-\mathrm{glycerides}+{\mathrm{CH}}_{3}\mathrm{OH}\frac{\mathrm{catalyst}}{\mathrm{Temperature}}\to \mathrm{Monoglycerides}+\mathrm{Methyl ester}$$

(3) $$\mathrm{Step}3\;\mathrm{Monoglycerides}+{\mathrm{CH}}_{3}\mathrm{OH}\frac{\mathrm{catalyst}}{\mathrm{Temperature}}\to \mathrm{Methyl ester}+\mathrm{Glycerol}$$
